# Transposition of Isolated Left Vertebral Artery in Hybrid Thoracic Endovascular Aortic Repair

**DOI:** 10.3389/fcvm.2021.783656

**Published:** 2021-12-14

**Authors:** Guangmin Yang, Hongwei Chen, Guangxiao Sun, Wensheng Lou, Xin Chen, Leiyang Zhang

**Affiliations:** Department of Thoracic and Cardiovascular Surgery, Nanjing First Hospital, Nanjing Medical University, Nanjing, China

**Keywords:** hybrid technique, thoracic endovascular aortic repair, isolated left vertebral artery, bypass, transposition

## Abstract

**Objectives:** The aim of this study was to present our experience with the management of isolated left vertebral artery (ILVA) during complex thoracic aortic pathology treated with the hybrid thoracic endovascular aortic repair.

**Methods:** This is a single-center, respective cohort study. Between June 2016 and June 2020, 13 patients (12 men; median age 60 years old, range 42–72 years old) who underwent hybrid procedures were identified with ILVA in our center. Demographics, imaging features, operation details, and follow-up in these patients were collected and analyzed.

**Results:** In this study, all patients received the hybrid procedure, and the primary technical success rate was 100%. There were no in-hospital deaths. Complication occurred in two (15.4%) patients. One patient suffered from contrast-induced acute kidney injury (CI-AKI) and recovered before discharge. Another patient required reintervention for acute left-lower-limb ischemia, which was successfully treated using Fogarty catheter embolectomy. Immediate vagus/recurrent laryngeal never palsy, lymphocele, and chylothorax were not observed. The median duration of follow-up was 22 months (range, 13–29 months). No neurologic deficits, bypass occlusion, or ILVA occlusion or stenosis were observed during the follow-up. No aortic rupture, cerebrovascular accident, or spinal cord ischemia was observed during the follow-up period.

**Conclusions:** Our limited experience reveals that hybrid procedures [thoracic endovascular aortic repair (TEVAR), ILVA transposition, and left common carotid artery-left subclavian artery (LCCA-LSA) bypass] are relatively safe, feasible, and durable for the treatment of thoracic aortic pathology with ILVA. However, further technique durability and larger studies with long-term follow-up periods are warranted.

## Highlights

- **Type of research:** Single-center retrospective cohort study.
- **Key findings:** The 13 patients with type B aortic dissection (TBAD) and isolated left vertebral artery (ILVA) who underwent hybrid thoracic endovascular aortic repair (TEVAR) had excellent outcomes. The survival was 100% during a median follow-up period of 22 months (range, 13–29 months).
- **Take-home message:** Hybrid TEVAR is viable and relatively safe for patients with TBAD and ILVA.

## Introduction

Supra-aortic trunk (SAT) variation was common in the vertebral artery (VA). Isolated left vertebral artery (ILVA) has arisen directly from the aortic arch, usually between the left common carotid artery (LCCA) and the left subclavian artery (LSA), and it was the second most common anatomical variation of the SAT (1). The prevalence of this variation was 0.8–6.6% in patients with the thoracic aortic disease according to clinical studies (2–5). On encountering complex thoracic aortic pathology, to establish an adequate proximal landing zone in patients with ILVA, coverage of the LSA and VA was often necessary. Whereas, emerging data suggested that the reduction in flow through the left VA and LSA might increase the risk of postoperative stroke and spinal cord injury (SCI) (6, 7). Compared with the reconstruction of LSA, reconstruction of the ILVA brings more challenges to the vascular surgeon during thoracic endovascular aortic repair (TEAVR). Improper management of the ILVA might lead to posterior ischemia or infarction of the brain (8, 9). Coverage and revascularization of the LSA in type B aortic dissection (TBAD) patients with ILVA had been reported by previous case reports (6, 7, 10). However, because the cases were rare, there were no widely adopted strategies regarding the reconstruction of ILVA.

In this study, we reported our experience with hybrid treatment of thoracic aortic pathology with an ILVA and inadequate proximal landing zones, using the TEVAR and concomitant procedure.

## Materials and Methods

### Study Cohort

**Figure 1:**
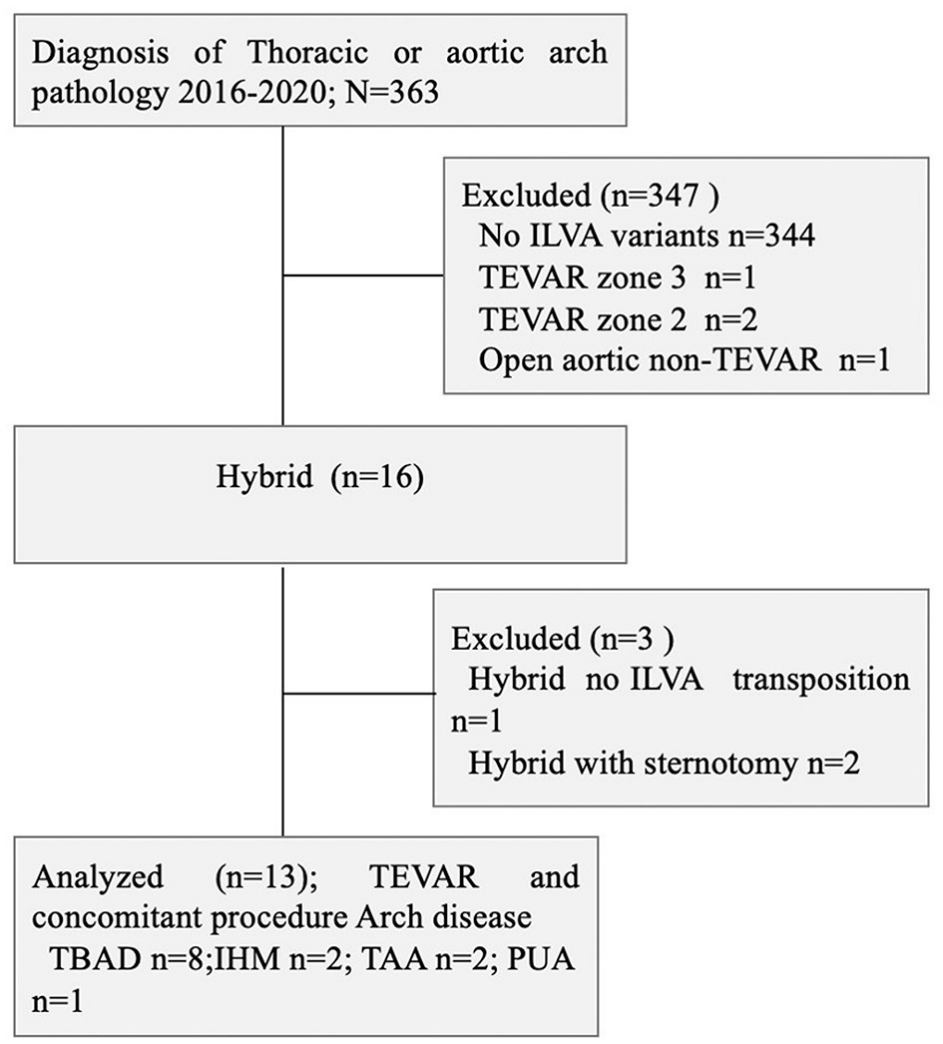
Consort diagram of thoracic aortic pathology repair (2016–2020; *n* = 363). ILVA, isolated left vertebral artery; TBAD, type B aortic dissection; IMH, intramural hematoma; TAA, thoracic aortic aneurysm; PUA, penetrating atherosclerotic ulcer; TEVAR, thoracic endovascular aortic repair.

This was a respective, single-center, and observational cohort study. From June 2016 to June 2020, there were 363 patients with the thoracic aortic disease who underwent TEVAR at the Department of Thoracic and Cardiovascular Surgery, Nanjing First hospital; 20 (5.5%) of them had complex thoracic pathology with ILVA. There were 13 patients who had preserved ILVA during TEVAR and concomitant procedure. There were eight TBAD (*n* = 8, TBAD), two intramural hematoma (*n* = 2, IMH), one penetrating atherosclerotic ulcer (*n* = 1, PAU), and two thoracic aortic aneurysms (*n* = 2, TAAs) (Figure 1). All those patients underwent aortic computed tomography angiography (CTA) and were diagnosed with the aortic disease with ILVA. The indications of intervention were defined according to recommended clinical practice Guidelines of the European Society for Vascular Surgery (ESVS) (11). Demographics, comorbidities, operation details, and early and late outcomes were collected and analyzed. The study involving human participants was reviewed and approved by the Local Ethics Committee of Nanjing First Hospital. This was a retrospective study; thus, informed consent was waived.

### Diagnosis and Definition

Zone 2 was divided into two parts in this study according to the previous Ishimaru zone classification (12). Zone 2a was defined as the proximal edge of the covered endograft that was distal to the LCCA but proximal to the ILVA origin; Zone 2b was defined as the proximal edge of the covered endograft that was distal to the ILVA but proximal to the LSA origin. All ILVA was diagnosed by CTA as the left VA arising directly from the aortic arch. Hypoplasia was defined as the cutoff VA diameter <2 mm (13). The primary technique success was defined as the successful deployment of the endograft with the exclusion of the aortic lesion without conversion to open surgery, type Ia or type III endoleak, operation-related death, and patency of the ILVA and LCCA-LSA bypass. Primary outcomes were early (<30 days) and late survival. The secondary endpoints were freedom from aortic-related mortality and ILVA patency during the follow-up.

### Surgical Technique

All procedures were performed in a hybrid operating room under general anesthesia. We routinely evaluated aortic arch characteristics, the geometry of SAT and carotid vessels, and integrity of Willis circle and both the vertebral arteries by digital subtraction angiography (DSA) before surgical procedure to exclude certain concomitant diseases or variants, which were undiscovered by CTA. All patients were diagnosed with thoracic aortic lesions with ILVA. To establish an adequate proximal landing zone in those patients, coverage of the LSA or VA was demanded.

### Transposition of ILVA and Bypass

We performed a single-stage intervention with transposition of ILVA and carotid-subclavian bypass (CSbp) to prevent potential proximal endoleaks. The transposition of the ILVA and the carotid-to-subclavian bypass were performed by a standard supraclavicular surgical access. The supraclavicular incision was made about 1 fingerbreadth width above the upper edge of the clavicle on the left side. An incision across the lateral head of the sternocleidomastoid muscle, extending an equal distance laterally, can expose both the subclavian artery and its branches, ILVA as well as the carotid artery. The left carotid artery was dissected free taking care to protect the vagus nerve. The VA dissection was continued and dissected free from beneath inside the LCCA until the fibrofatty tissue surrounding the ILVA was exposed. The subclavian artery was freed from beneath the clavicle to the proximal origin of the left internal mammary artery and thyrocervical trunk and taken care of to protect the phrenic nerve. Precautionary measures with respect to the lymphatics and nerves were needed and applied during the total procedure. Once the adequate length of the VA was exposed, a clamp was placed on the distal VA followed by the placement of vascular clips on the proximal aspect of the VA near its origin from the arch. Systemic heparin was given intravenously prior to placing the clamps. We then performed ligation on the VA in its proximal segment to have additional security. Next, the VA was traversed for anastomosis. A distal common carotid artery clamp was placed first followed by the placement of a proximal clamp. An arteriotomy was made with an 11 blade in the common carotid artery; once this was done, we continued and constructed an anastomosis between the VA and common carotid artery using 6-0 polypropylene sutures in an end-to-side manner using a running fashion. Once this anastomosis was constructed, the VA clamp was released first followed by a release of the distal common carotid and proximal common carotid artery clamp.

Subsequently, the subclavian artery was clamped and arteriotomy was prepared for anastomosis. The Gore vascular graft (WL Gore & Associates, Flagstaff, Arizona) was used to bridge the target vessels. The vascular graft (usually 8 mm) was anastomosed first to the LSA in an end-to-side manner, and the vascular graft was anastomosed sequentially to the common carotid artery in an end-to-side manner using 6-0 polypropylene sutures. The ILVA transportation was always transected as proximal as possible. It was essential to control the proximal stump after transecting before it was ligated. Cerebral oxygen saturation monitoring was performed during LCCA blocking period, and the anastomosis time was controlled within 10 min.

### The TEVAR Procedure

The stents graft main body was delivered through femoral artery access. Controlled hypotension and heart rate procedure were initiated before the deployment of endograft, with the systolic pressure controlled at around 90 mm Hg and the heart rate at 60–70 beats/min. For femoral artery access, we routinely performed the “pre-close technique” with two ProGlide devices (Abbott Vascular, Redwood City, CA) when patients' anatomy was favorable. Alternatively, surgical exposure of the femoral artery might be necessary. Access to the LSA can be gained *via* percutaneous puncture of the left brachial artery. A 5-F introducer sheath and catheter were then inserted so as to allow retrograde injection of contrast to delineate the anatomy and determine the site of stent graft deployment.

After accesses were obtained, a 5-F calibrated angiographic pigtail catheter was inserted for DSA to characterize the aortic pathology and the cerebral circulation, including the circle of Willis, vertebral arteries, and bilateral carotid arteries. Morphological features of the aortic arch lesions were measured on both the CTA and DSA images. Then, the extra stiff guidewire was advanced to the ascending aorta from the femoral artery for the thoracic main body stent graft. The thoracic stent graft was oversized by 0–20% based on indication for treatment (i.e., dissection, aneurysm, PUA, and IMH). The thoracic stent grafts were then deployed in the targeted landing zone. To coverage the primary lesion location and reduce type II endoleak. (Interlock; Boston Scientific, Natick, MA) were delivered at the origin of LSA through the retrograde catheter previously placed from the left branchial vessel. Retrograde hand injection of contrast was needed to evaluate the results of coils embolization.

### Postoperative Management

Unless contraindicated, patients were administered antiplatelet therapy (aspirin, 100 mg/day) indefinitely. Patients were monitored postoperatively with clinical and laboratory examinations. Head-and-neck CTA and magnetic resonance angiography (MRA) were needed when a cerebrovascular accident occurred. Upon suspicion of bypass and branch vessel malperfusion, additional angiography for further evaluation and possible reintervention was performed.

### Follow-Up

Patients were monitored closely with CTA imaging and laboratory examinations. CTA controls were performed at 1, 3, 6 months, and yearly thereafter. If the patient's condition is stable after 3 years of follow-up, the interval can be extended to 2–3 years. If patients demonstrate signs of adverse events or new symptoms, additional CTA or DSA examinations are performed. The collection of survival was completed either at the outpatient clinic visit or by telephone interviews.

### Statistical Analysis

Clinical data were prospectively recorded and analyzed. Categorical variables are presented as frequencies and percentages. Continuous variables are presented as mean ± SD and range.

## Results

### Study Cohort

**Figure 2:**
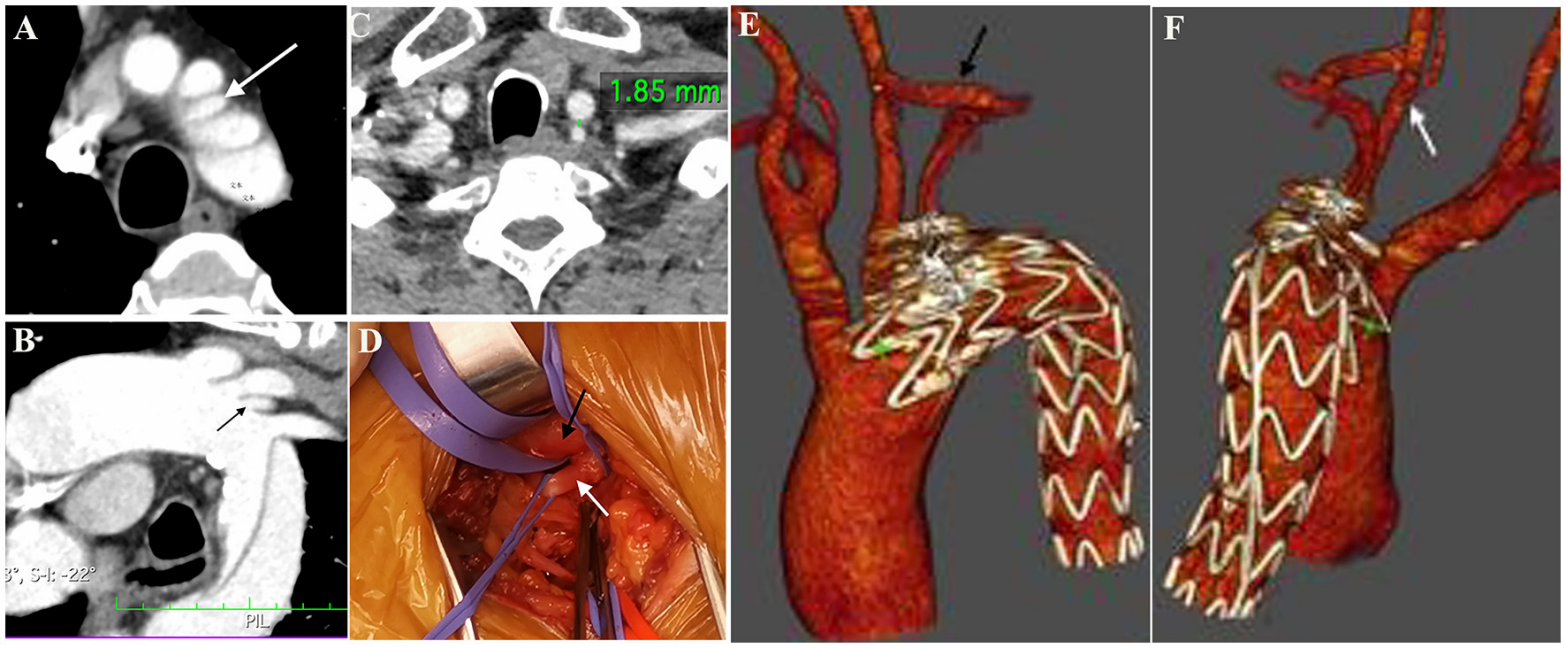
“Zone 2” TEVAR: Preoperative CTA showed the presence of a complex lesion of the descending aorta **(A)**. Multiplanar reconstruction documented the presence of an ILVA [**(B)**, white arrow] just proximal to the LSA origin. The distance between ILVA and LCCA was 1.85 mm **(C)**. Intraoperative view **(D)** showing the anatomy of LCCA (black arrow) and ILVA (white arrow). A Follow-up CTA study with the 3D volume confirmed the patency of the CSbp **(E)** and the transposed ILVA **(F)**. CTA, computed tomography angiography; LCCA, left common carotid artery; LSA, left subclavian artery; ILVA, isolated left vertebral artery; CSbp, carotid-subclavian bypass.

Between June 2016 and June 2020, there were 13 patients with thoracic aortic pathology with ILVA receiving hybrid procedures in our signal center (Figure 2). Twelve (92.3%) of the patients were men, and the median age was 61 years old (range, 42–72 years old). According to the diameter of the V1 segment of the VA, left VA dominance, symmetric VA, and right VA dominance accounted for three (23.1%), four (30.8%), and six (46.1%) of cases, respectively. The mean ILVA diameter was 4.81 ± 1.2 mm, and the right VA mean diameter was 5.09 ± 0.78 mm. Five (38.5%) cases of ILVA entered the circle of Willis to form the basilar artery, and eight (61.5%) cases of ILVA terminated at the posterior–inferior cerebellar artery (PICA).

Demographics, comorbidities, risk factors, and preoperative CTA imaging characteristics are shown in Table 1.

**Table 1:** Demography data, comorbidities, and imaging features.

**Variable**	**Median or No**.	**Range or %**
**Demography features**		
Age, years	60	42–72
Male sex	12	92.3
**Comorbidities**		
Hypertension	10	76.9
Dyslipidemia	9	69.2
COPD	5	38.5
Diabetes mellitus	6	46.1
Coronary artery disease	2	15.4
Previous stroke	12	92.3
Obesity (BMI > 30)	3	23.1
**Type of thoracic aortic disease**		
TBAD	8	61.6
TAA	2	15.4
IMH	2	15.4
PAU	1	7.7
**Dominance of vertebral artery**		
Right vertebral artery dominance	6	46.2
Symmetric vertebral artery	4	30.8
Left vertebral artery dominance	3	23.1
ILVA diameter, mm	5.09	3.2–6.15
**Thoracic aortic endografts (*****n*** **=** **13)**		
Length, mm	195	150–195

*COPD, chronic obstructive pulmonary disease; BMI, body mass index; TBAD, type B aortic dissection; TAA, thoracic aortic aneurysm; IMH, intramural hematoma; PUA, penetrating atherosclerotic ulcer; ILVA, isolated left vertebral artery*.

### Details of the Procedure

All patients underwent TEVAR, ILVA transposition, and LCCA-LSA bypass procedures. The aortic stent grafts were deployed in zone 2a. The median total operation time was 95 (range, 80–129 min) min, and the median ILVA blocking time was 8.3 min (range, 6.2–10.4 min). The main body stent grafts used in this procedure were the Gore (WL Gore & Associates, Flagstaff, AZ) TAG and relay thoracic graft (Bolton Medical, Sunrise, FL) devices. The median aortic coverage length was 195 mm (range, 150–195 mm). The mean length of stay in ICU after the operation was 1.5 ± 0.5 days, and the median hospitalization was 9 days (range, 7–13 days).

### Early and Late Outcomes

In this study, the primary technical success was achieved in all cases. There were no in-hospital deaths. Complications occurred in three patients. One patient suffered contrast-induced acute kidney injury (CI-AKI) with an increase in mean creatinine from a baseline of 58 umol/L to a peak at 228 umol/L and recovered before discharge. One case had incision hematoma after post-operation 12 h without dyspnea caused by tracheal hematoma compression and was treated conservatively. One patient required reintervention for acute left-lower-limb ischemia, which was successfully treated using Fogarty catheter embolectomy. Immediate vagus/recurrent laryngeal never palsy, lymphocele, and chylothorax were never observed. No aortic rupture, cerebrovascular accident, or spinal cord ischemia was observed in the early term.

The mean duration of follow-up was 22 months (range, 13–29 months). All patients who survived underwent a regular follow-up program. No neurologic deficits, bypass occlusion, or ILVA occlusion or stenosis were observed during the follow-up. We did not observe aortic-related mortality and aortic-related intervention. The outcomes are shown in Table 2.

**Table 2:** Early and late outcomes of thoracic aortic disease with ILVA after TEVAR and concomitant procedure.

**Variables**	**Median or No**.	**Range or %**
**Early outcomes**		
Technique success	13	100
CI-AKI	1	7.7
Spinal cord ischemia	0	0
Hematoma	1	7.7
Low extremity ischemia	1	7.7
Death	0	0
**Late outcomes**		
Follow-up, months	22	13–29
Vessel or bypass occlusion	0	0
Stroke	0	0
Unintended reintervention	0	0
Death	0	0

*CI-AKI, contrast-induced acute kidney injury*.

## Discussion

The normal VA joined to form the basilar artery and supply blood to the cerebellum and brain stem. The continuation of the left VA may also vary, including hypoplasia or termination in the PICA instead of merging with the basilar artery (1–5). About 1/3 of all ILVA cases were accompanied by PICA (14). The prevalence of a complete circle of Willis was 42%, while that was 89.7% in a general Western and Japanese population as reported, respectively, in studies (15, 16). However, compared with Western and Japanese populations, a complete circle of Willis was seen in only 27% of Chinese people in a recent report (17, 18). Misdiagnosis and mismanagement of the ILVA might lead to major neurologic complications if the circle of Willis was incomplete. Coverage of the LSA or VA was often necessary to establish an adequate proximal landing zone in patients with complex thoracic aortic pathology. While previously considered safe, emerging data suggest that the reduction in flow through the left VA by not revascularizing the LSA may increase the risk of postoperative stroke and SCI (6, 7, 10). A study by Ad et al. reported that the LCCA did not supply normal blood flow and the ILVA compensates for brain blood flow (19). Qi et al. stated that the ILVA should be reconstructed as soon as possible in patients with aortic dissection (20).

Although the importance of the contribution of the ILVA to brain circulation had been well-recognized, there were no clear guidelines because of the few data based on the description of case reports. Some previous studies had reported their early and late results of conventional open surgery and debranching surgery for aortic pathology with ILVA (18–21). Qi et al. (20) reported that 21 patients with aortic dissection (type A dissection, *n* = 20; type B dissection, *n* = 1) with an ILVA underwent total arch replacement with stented elephant trunk technique under hypothermic cardiopulmonary bypass. Two patients were subjected to SCI after implanted elephant trunk stent. There was one late death (4.8%) at a mean follow-up of 58 ± 16 months (range, 38–99 months). Piffaretti et al. (21) presented their experience of nine patients with aortic arch pathologies managing the ILVA during the hybrid/debranching aortic arch repair. Transposition of the ILVA was selectively performed in six patients. Two patients performed open ascending/arch repair with ILVA transposition; four patients underwent ILVA transposition with concomitant CSbp and TEVAR completion. One patient occurred retrograde acute type A dissection after type I hybrid aortic arch repair. Horner's syndrome was observed in one patient. The median follow-up was 15 months (range, 3–72 months); one patient died after 4 months due to respiratory insufficiency. Ding et al. (22) also reported their experience with 31 patients of type of B dissection with an ILVA. They preferred to use a chimney stent to revascularize the SAT in avoiding different complications. They selectively preserved dominant left VA in two cases using “zone 3” TEVAR. The technique success rate was 96.8% (30/31), and one (3.2%) showed immediate-type Ia endoleak. One (3.2%) had puncture-related complications due to femoral artery pseudoaneurysm. One (3.2%) case experienced transient spinal cord ischemia but gradually recovered. During the median follow-up of 33 months (range, 2–90 months), one (3.2%) required reintervention due to type II endoleak by sealing of the origin of the LSA with coils. Although open surgery and the debranching repair had encouraging outcomes in managing the patients with ILVA, elderly or high-risk patients may not be able to endure median sternotomy and fatal complications.

Endovascular intervention in thoracic aortic disease had become the mainstream treatment due to its less-invasive and promising perioperative outcomes in the past two decades. The parallel or fenestrated/branched technique was recommended as a valuable option for the treatment of complex thoracic aortic pathology. However, using parallel or fenestrated/branched techniques were thought to potentially risk of an endoleak due to a gutter formation. To avoid this dilemma, we had taken a hybrid technique, including TEVAR and concomitant procedure (ILVA transposition, LCCA-LSA bypass), in patients with complex thoracic aortic pathology with ILVA. Compared with the traditional hybrid/debranching technique and total endovascular aortic repair, the new hybrid techniques (TEVAR and concomitant procedure) had the advantages of improved safety and reduced invasiveness and branch instability by avoiding hypothermic circulatory arrest and aortic cross-clamping. In our study, there were no complications such as wound bleeding, wound infection, bypass occlusion, and local nerve injury. The technique success was 100% (13/13); vagus/recurrent laryngeal nerve palsy, cerebrovascular accident, or SCI was not observed. To our knowledge, this study was the largest series on hybrid technique without median sternotomy in thoracic aortic pathology with an ILVA with mid-term outcomes.

For this specific issue of ILVA, its management during thoracic aortic surgery had been controversial. Given a high incidence of an incomplete circle of Willis in the Chinese population (17, 18), we preserve routinely the ILVA using the hybrid technique in our study. On encountering an ILVA, further head-and-neck contrast-enhanced CTA prior to aortic repair was required to assess the dominance of vertebral arteries and the integrity of the circle of Willis. In this study, the incidence of ILVA was 3.6% (13 of 363) in patients with aortic pathologies using TEVAR and concomitant procedure. Six (46.25) of cases in our patient cohort were right VA dominant, four (30.8%) of cases were symmetric VA, whereas left VA dominance accounted for only three (23.1%) of cases. Although the presence of an ILVA was not associated with any clinical symptoms, it may be associated with the risk of SCI and infarction of the brain after the TEVAR procedure. Our preference for ILVA reconstruction was the transposition of the artery onto the carotid artery. This technique preserves maximally the VA in patients with normal anatomy and avoids the prosthetic of carotid to ILVA. We favor the preservation of VA that ends in a PICA with the diameter > 2 mm or VA joined to form the basilar artery, to avoid posterior circulation ischemia or potential risk of SCI. Furthermore, the technical aspects were no less important than the operation planning process. The technical aspects were concluded as follows: (1) in general, the LSA was easily exposed by the supra-clavicular approach and the ILVA was accessible due to its posterior, parallel course to the LCCA. (2) The technique was feasible when the vertical distance between the ILVA and the LCCA was <1 cm; otherwise, the expose of ILVA was difficult technically in most cases. (3) One small incision of supraclavicular surgical access was feasible and safe during ILVA transposition and LCCA-LSA bypass procedure. In our experience, we performed ILVA transposition first followed by LCCA-LSA bypass. This sequence allowed antegrade perfusion to the ILVA during carotid clamping for LCCA-LSA bypass, thus minimizing the risk of ischemic complications.

Nevertheless, many obvious limitations exist in our study. First, the study is observational and retrospective, the cohort is small, and there was no control group. Second, the duration of the follow-up was relatively short. These features and the limited experience of such anatomic variants do not allow us to make definitive recommendations from a clinical point of view. We hope that future larger registries or studies will help to better address such a challenging situation of surgical management of such a clinical scenario.

## Conclusion

Our limited experience reveals that hybrid procedures (TEVAR, ILVA transposition, and LCCA-LSA bypass) are relatively safe, feasible, and durable for the treatment of thoracic aortic pathology with ILVA. Further studies with a larger sample of patients and longer follow-up are needed to confirm this finding.

## Data Availability Statement

The original contributions presented in the study are included in the article/supplementary material, further inquiries can be directed to the corresponding author/s.

## Ethics Statement

The studies involving human participants were reviewed and approved by the Local Ethics Committee of Nanjing First Hospital. Written informed consent was not required for this study, in accordance with the local legislation and institutional requirements.

## Author Contributions

All authors listed have made a substantial, direct, and intellectual contribution to the work and approved it for publication.

## Conflict of Interest

The authors declare that the research was conducted in the absence of any commercial or financial relationships that could be construed as a potential conflict of interest.

## Publisher's Note

All claims expressed in this article are solely those of the authors and do not necessarily represent those of their affiliated organizations, or those of the publisher, the editors and the reviewers. Any product that may be evaluated in this article, or claim that may be made by its manufacturer, is not guaranteed or endorsed by the publisher.
